# Sequential targeted therapy for anaplastic lymphoma kinase-positive diffuse large B-cell lymphoma: a case report

**DOI:** 10.3389/fmed.2026.1932586

**Published:** 2026-09-22

**Authors:** Guocheng Zhong, Yiran Wang, Tian Zhang, Wei-Jie Liao

**Affiliations:** 1 Department of Hematology and Oncology, International Cancer Center, Shenzhen Key Laboratory of Precision Medicine for Hematological Malignancies, Shenzhen University General Hospital, Shenzhen University Clinical Medical Academy, Shenzhen University Health Science Center, Shenzhen, China; 2 Tsinghua Shenzhen International Graduate School, Tsinghua University, Shenzhen, China

**Keywords:** alectinib, ALK inhibitor, ALK+ DLBCL, brigatinib, case report, ensartinib, lorlatinib

## Abstract

**Background:**

Anaplastic lymphoma kinase-positive diffuse large B-cell lymphoma (ALK + DLBCL) is a rare and highly aggressive mature B-cell lymphoma with poor responses to conventional chemotherapy and no established standard treatment. Although ALK inhibitors have shown potential activity in ALK-rearranged malignancies, their clinical utility and optimal sequencing in ALK + DLBCL remain unclear.

**Case presentation:**

We report a 41-year-old male patient diagnosed with ALK + DLBCL harboring a TFG-ALK rearrangement. The patient experienced progressive disease during the clinical course and showed rapid, although transient, responses to sequential ALK inhibitor therapy. Subsequent chemotherapy-based salvage regimens provided only limited disease control. The patient received sequential ALK-directed therapy with alectinib, ensartinib, and lorlatinib, followed by a short brigatinib rechallenge before transplantation. These ALK inhibitor-based treatments provided intermittent disease control over more than one year. After lorlatinib followed by combination chemotherapy and a short brigatinib rechallenge, the patient proceeded to hematopoietic stem cell transplantation (HSCT). However, the disease relapsed four months after transplantation, with rapid and extensive bilateral pulmonary involvement, ultimately leading to respiratory failure and death.

**Conclusion:**

This case highlights both the clinical utility and limitations of sequential ALK inhibitor therapy in ALK + DLBCL. A short brigatinib rechallenge was also attempted before transplantation, although its objective efficacy could not be established because of the limited treatment duration. Sequential targeted therapy may induce rapid tumor regression and help bridge patients to HSCT; however, responses are not durable, resistance can emerge rapidly, and post-transplant relapse may remain highly aggressive. More effective combination or consolidation strategies are needed to improve long-term outcomes in this rare lymphoma subtype.

## Introduction

Anaplastic lymphoma kinase-positive diffuse large B-cell lymphoma (ALK + DLBCL) is a rare and aggressive subtype of mature B-cell lymphoma. Since it was first described in 1997, ALK + DLBCL has been recognized as a distinct clinicopathological entity and is now included in modern classifications of hematolymphoid tumors (1, 2). This disease accounts for less than 1% of diffuse large B-cell lymphomas and is characterized by ALK protein expression driven by ALK rearrangements, most commonly CLTC-ALK, although other fusion partners have also been reported (2–5).

ALK + DLBCL is clinically aggressive and diagnostically challenging. Patients frequently present with advanced-stage disease, extranodal involvement, elevated lactate dehydrogenase levels, and rapidly progressive tumor burden (3–6). Histologically, the tumor often shows immunoblastic or plasmablastic morphology and may mimic plasmablastic lymphoma or other aggressive lymphoid malignancies. Immunophenotypically, tumor cells usually express ALK and plasma cell-associated markers, whereas mature B-cell markers such as CD20 are often absent or weakly expressed (5, 6). Therefore, the diagnosis requires integrated assessment of morphology, immunophenotype, molecular findings, and, in difficult cases, expert pathological review.

The optimal treatment strategy for ALK + DLBCL remains undefined. Conventional chemotherapy regimens, including CHOP, CHOEP, EPOCH, and other intensified approaches, have generally produced unsatisfactory outcomes, especially in patients with advanced-stage, relapsed, or refractory disease (3, 4, 6). ALK + DLBCL is associated with poor prognosis, with a reported 5-year overall survival rate of approximately 28% (6). These findings highlight the need for more effective therapeutic strategies beyond conventional chemotherapy.

Because ALK rearrangement represents a key oncogenic driver, ALK inhibition is a biologically rational therapeutic approach for ALK + DLBCL. Early experience with crizotinib suggested potential activity but limited durability in some cases (7). More recently, next-generation ALK inhibitors, including alectinib and lorlatinib, have shown encouraging activity in relapsed or refractory ALK + DLBCL, including rapid tumor regression in preclinical models and clinical responses in patients (8, 9). However, the evidence remains limited, and the role of sequential ALK inhibitor therapy, particularly as a bridge to hematopoietic stem cell transplantation (HSCT), has not been well established.

Here, we report a rare case of TFG-ALK-rearranged ALK + DLBCL with progressive disease during a complex treatment course. The patient achieved rapid but transient clinical responses to sequential ALK inhibitor therapy with alectinib, ensartinib, and lorlatinib, which provided temporary disease control and created a therapeutic window for subsequent HSCT. This case provides clinical evidence that sequential ALK inhibition may transiently control TFG-ALK-rearranged ALK + DLBCL and allow transition to HSCT, while rapid resistance remains a major barrier to durable remission.

## Case presentation

A 41-year-old male patient was referred to our hospital with a history of recurrent cervical masses for more than two years and was ultimately diagnosed with ALK + DLBCL. In August 2020, he first noticed painful left cervical lymph node enlargement. Cervical ultrasound revealed hypoechoic nodules in regions III–IV of the neck. Biopsy at that time showed lymphoid tissue hyperplasia with focal atypical lymphoid cells, and malignancy could not be excluded. In December 2020, new painful cervical masses appeared on the right side. On March 15, 2021, he underwent maxillofacial and neck surgery for mass resection, and pathological examination again revealed atypical cells suggestive of neoplasm. These early pathological examinations were inconclusive and did not establish a definitive lymphoma diagnosis.

On May 27, 2021, positron emission tomography/computed tomography (PET/CT) demonstrated multiple hypermetabolic lesions suspicious for malignant lymphoma, with increased bone marrow glucose metabolism considered to represent reactive changes (Figure 1A). Targeted NGS was performed by a commercial clinical laboratory (Burning Rock Biotech, Guangzhou, China) using a 168-gene capture-based sequencing panel (Supplementary Table 1). The analysis identified a TFG-ALK rearrangement with a variant allele frequency of 14%, with no other clinically relevant genomic alterations detected. The tumor mutation burden was low (5.76 mutations/Mb), and microsatellite status was stable. Because a definitive pathological diagnosis of lymphoma had not yet been established, the patient received toripalimab plus temozolomide beginning on May 29, 2021; however, durable disease control was not achieved. Follow-up PET/CT on October 19, 2021 showed progressive disease (Figure 1B). Given the presence of the TFG-ALK rearrangement and continued disease progression, alectinib was initiated at a dose of 600 mg twice daily. After one month of treatment, the superficial tumor masses markedly decreased in size. Stable disease was observed on January 6, 2022. However, the patient subsequently developed worsening shortness of breath and newly enlarged left axillary lymph nodes. PET/CT on February 10, 2022 confirmed disease progression (Figure 1C), and chest CT further demonstrated rapid disease progression after acquired resistance to alectinib (Supplementary Figure 1).

**Figure 1:**
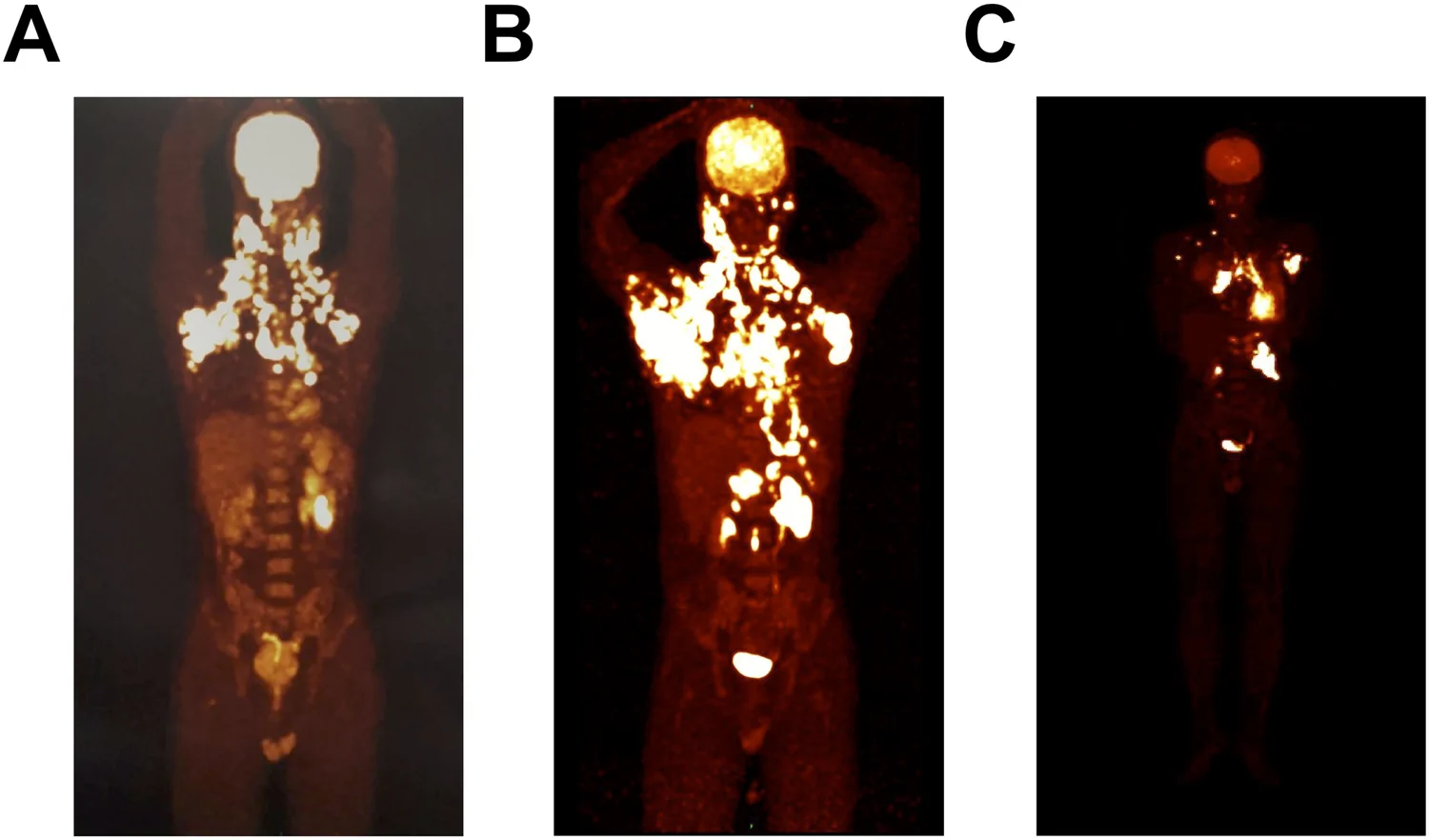
PET/CT images before and after alectinib treatment. **(A)** Two months after maxillofacial and neck surgery; **(B)** Before treatment with alectinib; **(C)** After treatment with alectinib for three months.

During subsequent disease progression, several non-ALK-directed salvage treatments were attempted, including radiotherapy, anti-angiogenic therapy, albumin-bound paclitaxel, decitabine, and DC-CIK cell infusion. These interventions provided only limited symptomatic relief and failed to achieve durable disease control. Follow-up imaging on May 9, 2022 showed further disease progression (Figures 2A,B). Repeat targeted sequencing of a mediastinal lesion revealed persistence of the TFG-ALK fusion with an increased variant allele frequency of 26.1%, together with an acquired ALK p.I1171N mutation at a variant allele frequency of 15%. The tumor mutation burden had increased to 13.44 mutations/Mb, while microsatellite status remained stable.

**Figure 2:**
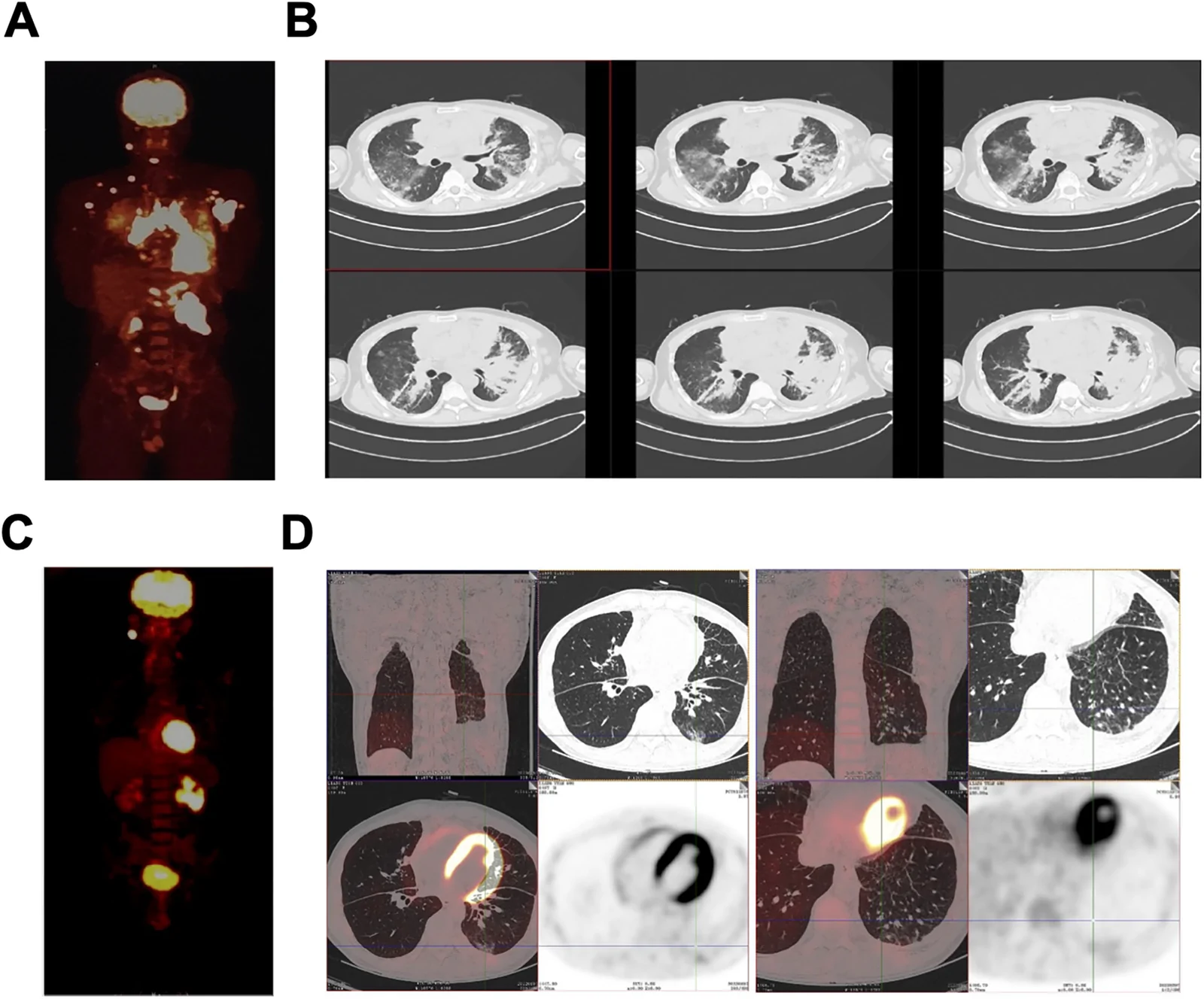
PET/CT images before and after treatment with ensartinib. **(A)** PET image before treatment with ensartinib; **(B)** Chest CT image before treatment with ensartinib; **(C)** PET image after treatment with ensartinib; **(D)** Chest CT image after treatment with ensartinib.

Given the persistent ALK rearrangement and acquired resistance to alectinib, ensartinib was initiated at a dose of 225 mg once daily. The patient experienced rapid clinical improvement after ensartinib treatment, with reduction in heart rate, alleviation of dyspnea, and shrinkage of submental, cervical, and left axillary tumor masses (Figures 2C,D). Treatment-related adverse events, including rash and liver function impairment, were observed but were tolerable. In mid-June 2022, the patient developed enlargement of a right cervical mass. PET/CT showed isolated progression in the right cervical region (Figure 3A), and surgical resection was performed. Histopathological examination demonstrated diffuse infiltration by medium-to-large atypical lymphoid cells with frequent mitotic figures, accompanied by focal necrosis and fibrosis. Immunohistochemical staining showed diffuse expression of CD45 and ALK in the atypical lymphoid cells, together with positivity for the B-cell-associated transcription factors PAX5, OCT2, and BOB1. A subset of tumor cells expressed EMA, whereas CD138 and MUM1 were weakly positive in only a small proportion of cells. CD30 and CD10 were negative, and the Ki-67 proliferation index was approximately 70%. (Figure 3B and Supplementary Table 2). B-cell monoclonality analysis revealed IgVH and IgK gene rearrangements (Supplementary Table 3), whereas no T-cell receptor rearrangement was detected (Supplementary Table 4). Based on the morphological, immunophenotypic, molecular, and clonality findings, the definitive diagnosis of ALK-positive large B-cell lymphoma was established following expert pathological consultation in August 2022.

**Figure 3:**
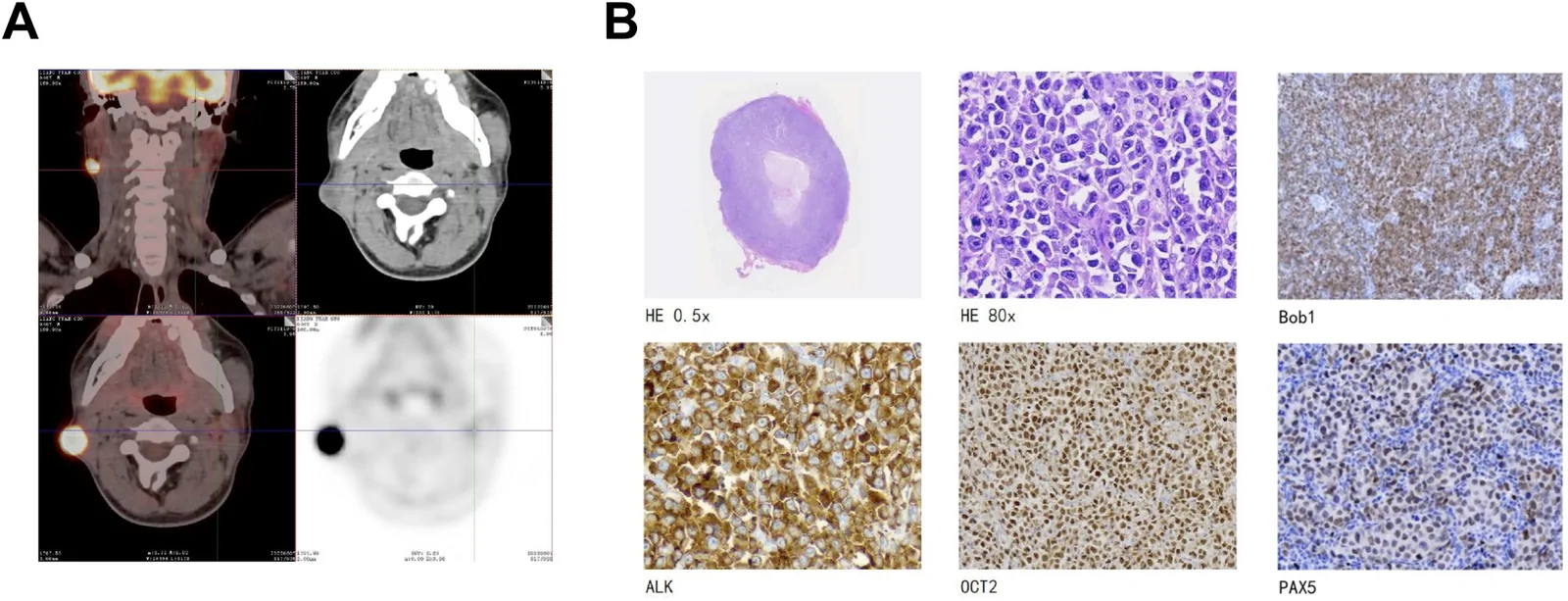
PET/CT images and IHC staining of the tumor mass on the right side of the neck. **(A)** Representative PET/CT images. **(B)** IHC staining of tumor sections, H&E staining at 0.5 × and 80 ×, ALK, OCT2, BOB1, and PAX5 staining.

On August 6, 2022, follow-up PET/CT again demonstrated progressive disease. Bone marrow examination, flow cytometry, cytogenetic analysis, and repeat tumor assessment did not reveal additional chromosomal abnormalities or new ALK rearrangements. The patient subsequently received conventional chemotherapy-based salvage regimens, including CHOP-E combined with lenalidomide and dose-adjusted EPOCH combined with lenalidomide. Although transient symptom relief was achieved, the disease remained refractory and progressed rapidly. On October 12, 2022, PET/CT confirmed further disease progression (Figure 4A). Because the patient was initially unable to tolerate conventional chemotherapy because of his poor clinical condition and high tumor burden, lorlatinib was initiated on October 13, 2022. Marked clinical improvement and tumor reduction were observed within approximately one week, after which dexamethasone, ifosfamide, cisplatin, and etoposide (DICE) chemotherapy was administered once the patient's condition had improved sufficiently to tolerate cytotoxic treatment. Exertional dyspnea significantly improved, serum lactate dehydrogenase levels gradually decreased, and PET/CT on December 24, 2022 demonstrated marked disease remission (Figure 4B).

**Figure 4:**
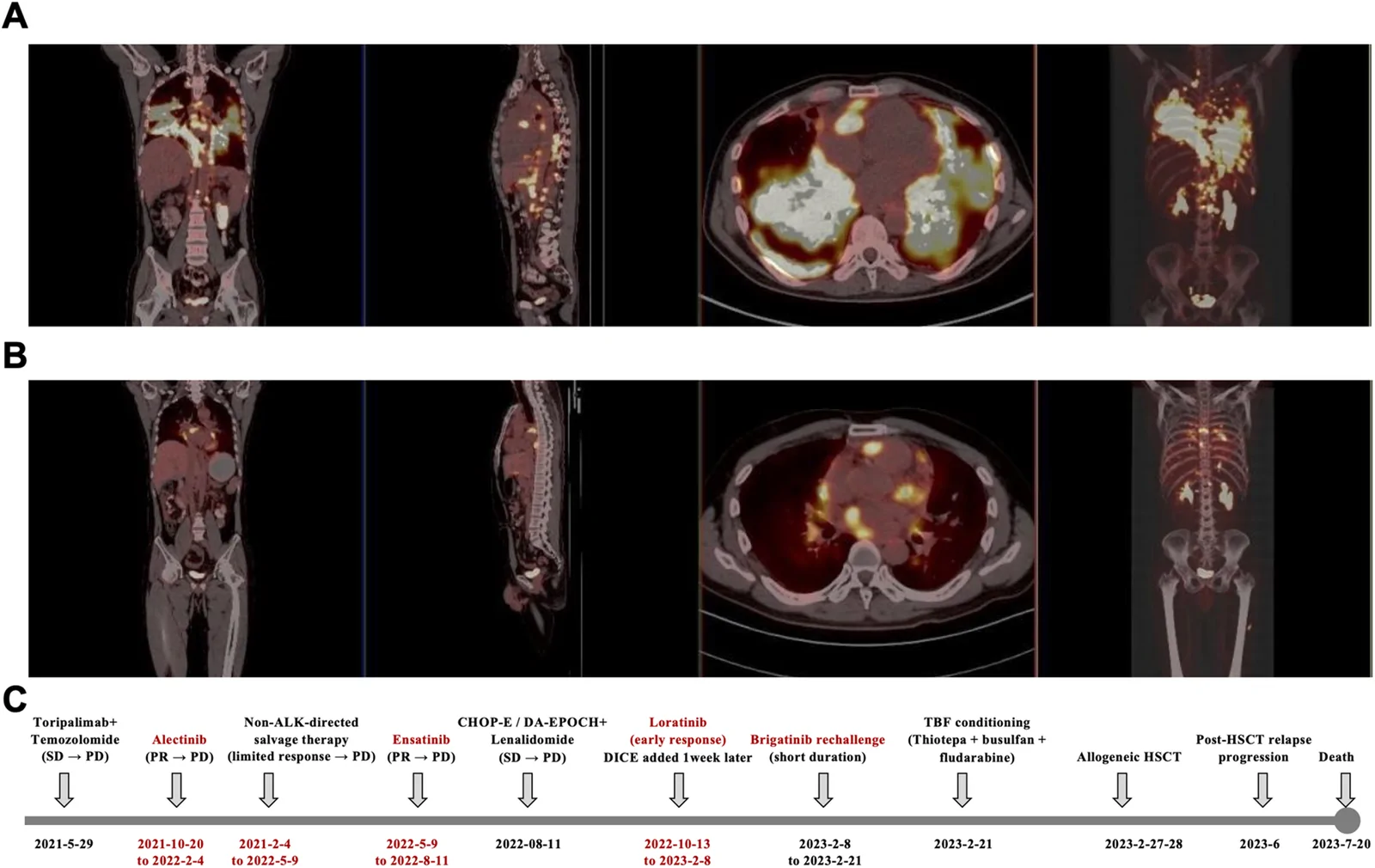
PET/CT images before and after lorlatinib-based treatment and summary of the clinical course. **(A)** PET image before treatment with lorlatinib; **(B)** PET image after lorlatinib followed by DICE chemotherapy. **(C)** A visual schema summarizing the timeline of the patient's clinical course.

Despite this response, the disease remained difficult to control. Follow-up blood-based NGS again detected the TFG-ALK fusion, ALK p.I1171N mutation, ALK intergenic rearrangement, ALK p.R1192P variant, and LRP1B p.A605D variant. On February 8, 2023, brigatinib, a second-generation ALK inhibitor, was initiated as an exploratory ALK-targeted rechallenge after failure of the preceding treatment lines. Initial symptomatic improvement was observed; however, the patient subsequently developed significant bilateral shoulder muscle pain. Given the refractory nature of the disease, poor response to conventional chemotherapy, repeated emergence of resistance to ALK inhibitors, and high tumor burden, salvage allogeneic hematopoietic stem cell transplantation (HSCT) was pursued after extensive discussion with the patient and his family.

From February 21, 2023, the patient underwent pre-transplant conditioning with the thiotepa, busulfan, and fludarabine (TBF) regimen. For graft-vs.-host disease (GVHD) prophylaxis, cyclosporine A (CsA), methotrexate (MTX), mycophenolate mofetil (MMF), and antithymocyte globulin (ATG; 6 mg/kg) were administered. High-resolution HLA typing confirmed the patient's son as a haploidentical related donor, and peripheral blood hematopoietic stem cells were infused on February 27 and 28, 2023 (Supplementary Table 5), with a total infusion volume of 170 mL. The infused mononuclear cell and CD34 + cell doses were approximately 4.93 × 10^8/kg and 4.92 × 10^6/kg, respectively. The transplantation procedure was uneventful, with neutrophil engraftment achieved on day +11 and platelet engraftment on day +24 after transplantation. Donor chimerism assessed at approximately day +40 was greater than 80%, indicating mixed donor chimerism. After transplantation, serum lactate dehydrogenase (LDH) levels initially decreased but began to rise again from early April 2023, increasing from 235 U/L on April 5 to 1,897 U/L on May 17, in parallel with subsequent disease progression (Supplementary Figure 2). Four months after HSCT, the patient developed aggressive relapse with extensive bilateral pulmonary involvement. Repeat biopsy and molecular testing of an involved lymph node demonstrated persistence of the TFG-ALK fusion, although at a lower abundance than earlier in the disease course. The previously identified ALK p.I1171N resistance mutation also remained detectable. The disease subsequently progressed rapidly, resulting in respiratory failure and death.

## Discussion

ALK + DLBCL is an extremely rare and aggressive lymphoma subtype with limited evidence to guide optimal treatment. We describe a patient with TFG-ALK-rearranged ALK + DLBCL whose disease was poorly controlled by subsequent conventional chemotherapy-based salvage regimens, but showed rapid and transient responses to sequential ALK inhibitor therapy. The use of alectinib, ensartinib, and lorlatinib provided repeated periods of disease control and created a therapeutic window for subsequent allogeneic hematopoietic stem cell transplantation (HSCT). However, the patient ultimately developed aggressive post-transplant relapse, highlighting both the potential value and the major limitations of sequential ALK inhibition in this disease.

The diagnosis of ALK + DLBCL remains challenging because of its rarity and overlapping pathological features with other aggressive lymphoid malignancies. Since the first description of this entity by Delsol et al. in 1997, ALK + DLBCL has been recognized as a distinct clinicopathological disease characterized by ALK expression and frequent plasmablastic or immunoblastic morphology (2–5). Tumor cells may express plasma cell-associated markers and often lack or show weak expression of conventional mature B-cell markers such as CD20, which may lead to diagnostic confusion with plasmablastic lymphoma, ALK-positive anaplastic large cell lymphoma, or metastatic non-hematological malignancies (3, 5, 6). Accordingly, immunohistochemical evaluation, including ALK staining in an appropriate clinicopathological context, remains an important component of the diagnostic work-up for DLBCL and its uncommon variants (10). In our case, the diagnosis was supported by morphology, ALK positivity, expression of B-cell-associated transcription factors including PAX5, OCT2, and BOB1, molecular detection of TFG-ALK rearrangement, B-cell monoclonality with IgVH and IgK gene rearrangements, absence of T-cell receptor rearrangement, and expert pathological consultation. Recent studies have further highlighted the molecular heterogeneity of DLBCL, including noncoding alterations involving the PAX5/ZCCHC7 locus that can modify local regulatory activity and gene expression during lymphoma progression (11). These findings emphasize that an integrated diagnostic approach combining histopathology, immunophenotyping, clonality analysis, and molecular testing is essential for accurate diagnosis.

Treatment of ALK + DLBCL remains poorly defined. Conventional chemotherapy regimens used for aggressive B-cell lymphomas, including CHOP-like or EPOCH-based regimens, have generally shown limited efficacy in ALK + DLBCL, particularly in advanced-stage or relapsed/refractory disease (3, 6, 7). The frequent absence of CD20 expression may also limit the benefit of rituximab-containing regimens. In our patient, multiple chemotherapy-based and non-ALK-directed salvage treatments failed to achieve durable disease control, whereas ALK inhibition consistently produced rapid symptomatic and radiological improvement. This clinical pattern suggests that the lymphoma remained highly dependent on ALK signaling despite its aggressive and refractory behavior.

The clinical course of this patient suggests that different ALK inhibitors may retain activity after prior ALK inhibitor failure, although the duration of response was limited. The patient initially responded to alectinib, developed acquired resistance, and then responded again to ensartinib. Repeat sequencing after alectinib failure revealed persistent TFG-ALK fusion together with an acquired ALK p.I1171N mutation. Although the biological significance of this mutation in ALK + DLBCL remains uncertain, ALK I1171N has been reported as an alectinib-resistance mutation in ALK-rearranged lung cancer and may retain sensitivity to selected next-generation ALK inhibitors (12, 13). In this context, the response to ensartinib after alectinib resistance suggests incomplete cross-resistance among ALK inhibitors and supports repeat molecular profiling at disease progression to guide subsequent targeted therapy. Later, lorlatinib followed by DICE chemotherapy was associated with marked disease remission. Notably, clinical and tumor improvement was already observed after lorlatinib initiation and before DICE administration, although the respective contributions of lorlatinib and DICE to the final radiological response cannot be fully distinguished. Following lorlatinib-based treatment, brigatinib was subsequently attempted as an exploratory rechallenge with a second-generation ALK inhibitor. Although transient symptomatic improvement was observed, the treatment duration was short and no objective radiological response could be established before transplantation.

Nevertheless, the responses to ALK inhibitors were transient. Each period of clinical benefit was followed by rapid disease progression, and once resistance emerged, tumor burden increased quickly with severe cardiopulmonary compromise. This differs from the more established experience of ALK inhibitors in ALK-rearranged non-small-cell lung cancer, where durable disease control can often be achieved with modern ALK tyrosine kinase inhibitors. In ALK + DLBCL, available evidence remains limited to case reports and small series, although next-generation ALK inhibitors have shown encouraging activity in relapsed or refractory disease (8, 9). Our case adds to this evidence by showing that sequential ALK inhibition can serve as an effective temporary disease-control strategy, but it is unlikely to be sufficient as a definitive treatment.

In practice, the most meaningful role of ALK inhibition in this case was disease debulking before HSCT rather than definitive disease control. In this patient, sequential ALK inhibition reduced tumor burden and provided sufficient time to proceed to allogeneic HSCT. However, relapse occurred four months after transplantation with extensive bilateral pulmonary involvement, and the disease ultimately progressed to fatal respiratory failure. Post-transplant assessment showed mixed donor chimerism, with donor chimerism exceeding 80% at approximately day +40. At subsequent disease progression, repeat molecular analysis of an involved lymph node demonstrated persistence of the TFG-ALK fusion, although at a lower abundance than earlier in the disease course, together with persistence of the previously identified ALK p.I1171N resistance mutation. These findings indicate that transplantation did not completely eradicate the molecularly defined disease clone and may reflect ongoing clonal evolution under therapeutic pressure. Thus, even when ALK inhibitors successfully create a therapeutic window for transplantation, residual disease, clonal evolution, and rapid emergence of resistance may compromise long-term disease control. Therefore, for patients with relapsed or refractory ALK + DLBCL, ALK inhibitors may be best considered as part of a broader treatment strategy rather than as stand-alone therapy. The role of post-transplant ALK inhibitor maintenance also warrants consideration. In this patient, long-term ALK inhibitor maintenance was not administered after allogeneic HSCT, and aggressive relapse occurred approximately four months after transplantation. Post-transplant ALK inhibitor maintenance may represent a potential strategy for maintaining ALK suppression in selected patients; however, evidence in ALK-positive large B-cell lymphoma remains extremely limited, and no standard maintenance approach has been established. The optimal ALK inhibitor, timing of initiation, treatment duration, and potential interaction with post-transplant immune effects remain to be defined.

In this case, longitudinal serum lactate dehydrogenase (LDH) levels broadly paralleled changes in disease activity, with a marked decline during treatment response and a subsequent rise preceding overt post-transplant progression (Supplementary Figure 2). Although this observation is based on a single patient, LDH may serve as a readily accessible adjunctive marker for monitoring disease activity and treatment response in aggressive ALK + DLBCL. However, LDH is nonspecific and should not be interpreted in isolation; imaging and, where feasible, repeat molecular assessment remain important when disease progression or treatment resistance is suspected.

This report has several limitations. As a single case, it cannot establish the efficacy of a specific ALK inhibitor sequence or define the optimal timing of HSCT. Although an early clinical and tumor response was observed after lorlatinib initiation and before DICE chemotherapy was administered, the subsequent combination treatment makes it impossible to fully distinguish the respective contributions of lorlatinib and DICE to the final radiological remission. In addition, the mechanisms underlying repeated resistance were not completely characterized. Despite these limitations, this case provides clinically relevant evidence that molecularly guided sequential ALK inhibition can induce rapid responses and may bridge selected patients to HSCT, while also underscoring the urgent need for more durable strategies. Future studies should focus on systematic molecular monitoring at progression, rational sequencing of next-generation ALK inhibitors, combination approaches to delay resistance, and integration of ALK inhibition with cellular therapy or transplantation in this rare and aggressive lymphoma subtype.

## Conclusion

This case illustrates the clinical complexity of managing ALK + DLBCL and suggests that molecularly guided sequential ALK inhibition can provide meaningful but temporary disease control. In this patient, ensartinib remained active after acquired resistance to alectinib, and lorlatinib followed by DICE chemotherapy helped reduce tumor burden before allogeneic HSCT. A short brigatinib rechallenge was subsequently attempted, although its objective efficacy could not be established because of the limited treatment duration. However, the rapid emergence of resistance and aggressive post-transplant relapse indicate that ALK inhibition alone is unlikely to achieve durable disease control. Repeated molecular profiling at progression may help guide treatment selection, but more effective combination or consolidation strategies are needed for this rare and highly aggressive lymphoma subtype.

## Data Availability

The original contributions presented in the study are included in the article/Supplementary Material, further inquiries can be directed to the corresponding author.
