# Supplementary material for: Sequential targeted therapy for anaplastic lymphoma kinase-positive diffuse large B-cell lymphoma: a case report
Source: Front Med (Lausanne). 2026 Sep 22;13:1932586. doi: 10.3389/fmed.2026.1932586 (PMC13638396; doi:10.3389/fmed.2026.1932586)
Supplement: Supplementary file 2 [file Table1.docx]

**Supplementary Materials**

**Sequential targeted therapy for anaplastic lymphoma kinase-positive diffuse large B-cell lymphoma: a case report**

Guocheng Zhong^1^, Yiran Wang^1^, Tian Zhang^2^, Wei-Jie Liao^1,2, *^

**Correspondence:** Wei-Jie Liao: [liao-wj16@tsinghua.org.cn](mailto:liao-wj16@tsinghua.org.cn)

# 1. Supplementary Figure

**Figure S1. CT images indicated resistance to alectinib.**

(A) CT images after treatment with alectinib for about three months; (B) CT images after treatment with alectinib for about four months; (C) CT images after treatment with alectinib for about five months.

**Figure S2**. Longitudinal serum LDH levels during the clinical course. Serum LDH levels decreased markedly after lorlatinib followed by DICE chemotherapy and rose again after transplantation in parallel with disease progression. Vertical dashed lines indicate the initiation of lorlatinib, brigatinib, TBF conditioning, and allogeneic HSCT.

# 2. Supplementary Tables

**Table S1. Commercial targeted NGS panel**

| AKT1 | CCNE1 | EPHA3 | GATA2 | KIT | NFE2L2 | PPP2R1A | SOX2 |
| --- | --- | --- | --- | --- | --- | --- | --- |
| ALK | CD274 | EPHA5 | GATA3 | KMT2D | NOTCH1 | PRKDC | SOX9 |
| APC | CD74 | EPHA7 | GRIN2A | KRAS | NRAS | PTEN | SPOP |
| AR | CDH18 | EPHB1 | H3F3C | LRP1B | NTRK1 | PTPRD | SPTA1 |
| ARID1A | CDK4 | ERBB2 | HGF | MAP2K1 | NTRK2 | PTPRT | SRC |
| ATM | CDK6 | ERBB3 | HIST1H1C | MAP3K13 | NTRK3 | RAD50 | STAG2 |
| ATR | CDKN1A | ERBB4 | HIST1H3B | MAX | PAK7 | RAD51B | STK11 |
| B2M | CDKN1B | ESR1 | HIST1H3G | MCL1 | PALB2 | RAD51C | TBX3 |
| BARD1 | CDKN2A | FANCA | HRAS | MEN1 | PARK2 | RAD51D | TERT |
| BCL2L11 | CHEK1 | FANCI | IDH1 | MET | PARP1 | RAD54L | TGFBR2 |
| BCOR | CHEK2 | FAT3 | IDH2 | MLH1 | PDGFRA | RAF1 | TP53 |
| BLM | CREBBP | FBXW7 | IGF2 | MRE11A | PDGFRB | RARA | TP63 |
| BRAF | CSMD3 | FGF19 | IKZF1 | MSH2 | PIK3C2G | RB1 | TRIM58 |
| BRCA1 | CTNNB1 | FGF3 | IL7R | MSH6 | PIK3C3 | RBM10 | TRPC5 |
| BRCA2 | CYP2D6 | FGF4 | INHBA | MTOR | PIK3CA | RET | U2AF1 |
| BRINP3 | DIS3 | FGFR1 | JAK1 | MUTYH | PIK3CG | RNF43 | UGT1A1 |
| BRIP1 | DNMT3A | FGFR2 | JAK2 | MYC | PIK3R1 | ROS1 | VEGFA |
| CARD11 | DPYD | FGFR3 | KDM5A | MYCN | PMS2 | RUNX1 | VEGFB |
| CASP8 | EGFR | FLT1 | KDM6A | NAV3 | POLD1 | SETD2 | VEGFC |
| CBL | EMSY | FLT3 | KDR | NBN | POLE | SMAD4 | VHL |
| CCND1 | EP300 | FLT4 | KEAP1 | NF1 | POM121L12 | SMARCA4 | YES1 |

**Table S2. Immunohistochemical profile of the diagnostic cervical mass biopsy**

| Marker | Result |
| --- | --- |
| CD45 | Diffuse positive in atypical lymphoid cells |
| ALK | Positive in atypical lymphoid cells |
| BOB1 | Diffuse positive |
| OCT2 | Positive |
| PAX5 | Positive |
| EMA | Positive in a subset of tumor cells |
| CD138 | Weakly positive in a small proportion of tumor cells |
| MUM1 | Weakly positive in a small proportion of tumor cells |
| CD30 | Negative |
| CD10 | Negative |
| Ki-67 | Approximately 70% |
| CD20 | Positive in background B lymphocytes |
| CD79a | Positive in background B lymphocytes |
| CD3 | Positive in background T lymphocytes |
| CD5 | Positive in background T lymphocytes |
| S100 | Scattered positive cells |
| MelanA | Negative |
| HMB45 | Negative |

**Table S3. B-cell clonality assessment.**

| **Testing targets** | | **Result** |
| --- | --- | --- |
| Complete rearrangement of IgVH genes (V-D-J) | FR1-JH | **+** |
|  | FR2-JH | **+** |
|  | FR3-JH | **+** |
| Incomplete rearrangement of IgDH genes (D-J) | DH1-6-JH | **-** |
|  | DH7-JH | **-** |
| Rearrangement of IgK genes | Vκ-Jκ | **-** |
|  | Vκ-Kde+INTR-Kde | **+** |
| Rearrangement of IgL genes | Vλ-Jλ | **-** |

“+” represents Positive; “-” represents Negative

**Table S4. T-cell clonality assessment.**

| **Testing targets** | | **Result** |
| --- | --- | --- |
| Rearrangement of TCRB genes | TCRB A | **-** |
|  | TCRB B | **-** |
|  | TCRB C | **-** |
| Rearrangement of TCRG genes | TCRG A | - |
|  | TCRG B | - |
| Rearrangement of TCRD genes | TCRD | - |

“+” represents Positive; “-” represents Negative

**Table S5. High-resolution HLA typing results of the patient and donor.**

| HLA locus | Patient | Donor (son) | Shared allele |
| --- | --- | --- | --- |
| HLA-A | 02:07, 31:01 | 31:01, 32:01 | 31:01 |
| HLA-B | 46:01, 51:01 | 51:01, 52:01 | 51:01 |
| HLA-C | 01:02, 14:02 | 12:02, 14:02 | 14:02 |
| HLA-DRB1 | 11:01, 14:18 | 14:18, 15:02 | 14:18 |
| HLA-DQB1 | 05:03, 03:01 | 05:03, 06:01 | 05:03 |
| HLA-DPB1 | 02:02, 05:01 | 05:01, 14:01 | 05:01 |
